# Reporting of methods to prepare, pilot and perform data extraction in systematic reviews: analysis of a sample of 152 Cochrane and non-Cochrane reviews

**DOI:** 10.1186/s12874-021-01438-z

**Published:** 2021-11-06

**Authors:** Roland Brian Büchter, Alina Weise, Dawid Pieper

**Affiliations:** 1 Institute for Research in Operative Medicine (IFOM), Faculty of Health, School of Medicine, Witten/Herdecke University, Ostmerheimer Str. 200, 51109 Cologne, Germany; 2 Faculty of Health Sciences Brandenburg, Brandenburg Medical School Theodor Fontane, Institute for Health Services and Health System Research, Rüdersdorf, Germany; 3 Center for Health Services Research, Brandenburg Medical School Theodor Fontane, Rüdersdorf, Germany

**Keywords:** Systematic review methods, Evidence synthesis, Data extraction

## Abstract

**Background:**

Previous research on data extraction methods in systematic reviews has focused on single aspects of the process. We aimed to provide a deeper insight into these methods by analysing a current sample of reviews.

**Methods:**

We included systematic reviews of health interventions in humans published in English. We analysed 75 Cochrane reviews from May and June 2020 and a random sample of non-Cochrane reviews published in the same period and retrieved from Medline. We linked reviews with protocols and study registrations. We collected information on preparing, piloting, and performing data extraction and on use of software to assist review conduct (automation tools). Data were extracted by one author, with 20% extracted in duplicate. Data were analysed descriptively.

**Results:**

Of the 152 included reviews, 77 reported use of a standardized extraction form (51%); 42 provided information on the type of form used (28%); 24 on piloting (16%); 58 on what data was collected (38%); 133 on the extraction method (88%); 107 on resolving disagreements (70%); 103 on methods to obtain additional data or information (68%); 52 on procedures to avoid data errors (34%); and 47 on methods to deal with multiple study reports (31%). Items were more frequently reported in Cochrane than non-Cochrane reviews. The data extraction form used was published in 10 reviews (7%). Use of software was rarely reported except for statistical analysis software and use of RevMan and GRADEpro GDT in Cochrane reviews. Covidence was the most frequent automation tool used: 18 reviews used it for study selection (12%) and 9 for data extraction (6%).

**Conclusions:**

Reporting of data extraction methods in systematic reviews is limited, especially in non-Cochrane reviews. This includes core items of data extraction such as methods used to manage disagreements. Few reviews currently use software to assist data extraction and review conduct. Our results can serve as a baseline to assess the uptake of such tools in future analyses.

**Supplementary Information:**

The online version contains supplementary material available at 10.1186/s12874-021-01438-z.

## Background

Data extraction forms and the data extraction process are important foundations of any systematic review as they provide the basis for appraising, analysing, summarizing and interpreting the underlying evidence [1]. Adequate data extraction methods are important for several reasons.

Firstly, carefully developed and tested data extraction forms support high quality reporting of the features of primary studies in systematic review reports. This is crucial since it allows readers of the reviews to make sense of the underlying primary studies and assess their applicability to specific contexts. Previous studies have shown that many systematic reviews lack the information required to reproduce the interventions tested in the underlying studies [2]. Secondly, high quality extraction forms and bias reducing methods for data extraction reduce the risk of data extraction errors [3, 4]. Several studies have shown that these are frequent in systematic reviews and can lead to relevant changes to meta-analytic point estimates [5]. Thirdly, adequate data extraction methods produce a paper trail of data flow, judgements, and decisions. This increases transparency and replicability, and helps with possible corrections and updates of systematic reviews [6].

There is a large body of methodological surveys on the reporting quality and characteristics of systematic reviews. These studies have typically used a broad approach and have looked at single aspects of the data extraction process – most notably the number of reviewers involved in data extraction [7–12]. Some replication of such studies seems justified, for example to track reporting quality over time. Given the amount of this literature, however, it seems prudent to provide deeper insights into specific aspects of the systematic review process instead of producing an increasing number of studies broadly analysing adherence to reporting guidelines or methodological quality. A significant number of decisions, judgements, assumptions, and simplifications may be needed in the systematic review process and these require appropriate methods. Many of these are directly linked to the choices for data extraction methods and tools [6]. These can include, for example, questions about clinical issues, different outcome measures, reporting and multiplicity of outcomes, dealing with multiple study reports and avoiding double counting [13].

Furthermore, an increasing number of (semi-)automation tools to support systematic review conduct are now available. These have the potential to improve workflows, save time, increase transparency and reproducibility, and reduce risk of errors [14, 15]. There are also possible downsides, for example by limiting flexibility or encouraging potentially inappropriate shortcuts [3, 16]. Collecting information on the prevalence of use of these tools will help to assess how they are currently accepted by systematic reviewers. It also provides a baseline to track future uptake.

Despite the importance of the data extraction process, we are not aware of any in-depth analyses of the methods used to prepare, pilot and perform data extraction in systematic reviews in the health care field. The aim of this project was to fill this gap by analysing these aspects of data extraction in a current sample of Cochrane and non-Cochrane systematic reviews, including the use of software to support the conduct of systematic reviews.

## Methods

This study protocol for this project was published a priori in the Open Science Framework (https://osf.io/ekt94/).

### Study sample

We aimed to include 75 Cochrane Reviews and 75 non-Cochrane reviews for analysis. The sample size was based on previous methodological studies on systematic review methods [17–19].

For the Cochrane Reviews we linked the most recent iteration of the review with the protocol. For non-Cochrane reviews we linked the reviews with both protocols and records from study registries (e.g., PROSPERO) and analysed these together. We included protocols irrespective of their publication status, i.e., whether they were published in a journal, as a supplement to the review or in a repository. We did not conduct extra searches to identify protocols or study registry entries. This decision was based on the assumption that most authors are likely to refer to study protocols or registrations in their publication. When authors reported that a protocol was available on request, we contacted them to retrieve it. We excluded protocols not published in English.

### Eligibility criteria

To be eligible for inclusion, non-Cochrane reviews had to fulfil the following criteria for a systematic review based on a modified definition from Krnic Martinic and colleagues [20]:

- the research question was clearly defined
- the search sources were reported (minimum requirement: reporting of the bibliographic databases that were searched)
- the eligibility criteria were reported
- the study selection methods were reported
- the included studies were assessed for methodological limitations/risk of bias and the appraisal methods and results were reported

Furthermore, we restricted our analysis to systematic reviews of medical treatments in humans published in English, irrespective of the design of the studies included in the reviews.

We excluded journal publications of Cochrane reviews to avoid biasing comparisons across review types. We also excluded systematic reviews on SARS-CoV-2 or Covid-19 based on the assumption that these may be less representative of the methods typically applied due to the urgent nature of the pandemic.

### Search methods

First, we retrieved the sample of Cochrane reviews and their protocols from the Cochrane Database of Systematic Reviews via the Cochrane Library. We use a backward consecutive sample of the last 75 Cochrane Reviews that were published starting with issue 6, 2020 (the most current issue at the time the searches for this project were conducted). Cochrane reviews were retrieved by one author (RBB).

We then conducted a search for non-Cochrane reviews published in the same time period as the Cochrane Reviews. These were retrieved from Medline via PubMed. To identify non-Cochrane reviews we combined a specific filter for systematic reviews and a sensitive filter for treatment studies [21, 22]. The search was also combined with search terms relevant to SARS-CoV-2 or Covid-19 using the boolean operator “not” to exclude this topic from the search results. The full search strategy is reported in Additional file 1.

We imported the search results from the Medline search to EndNote and copied the formatted references into a Microsoft Excel spreadsheet, where we used the random function to bring them in a random order. We then screened them for eligibility consecutively until a random sample of at least 75 eligible systematic reviews was reached.

Two of us (RBB and AW) independently screened the titles and abstracts of the references on the list until each of us had identified 100 potentially relevant systematic reviews. We discussed disagreements on inclusion until consensus was reached and, if necessary, included a third reviewer for arbitration (DP). We then retrieved the full texts of the potentially relevant reviews and assessed them against the full inclusion criteria. Again, this was done by two reviewers independently (RBB, AW). Disagreements on final inclusion were discussed to reach a consensus. A third reviewer acted as an arbiter if agreement could not be reached (DP). As the first 100 potentially relevant articles were not sufficient to attain the planned sample size, we continued to screen the list in bouts of 20 references using the same methods until the sample was full. We developed a study selection form to document whether the retrieved full texts meet the eligibility criteria (Additional file 2). This was also used to collect information on the review characteristics such as the country of the corresponding author and the number of included studies.

During title and abstract screening, we noticed some disagreements due to a different understanding of what constitutes a medical intervention. After discussion we agreed to consider all types of interventions that can be initiated or encouraged by a health care professional (including e.g., exercise, dietary interventions and psychological interventions but excluding e.g., interventions to increase uptake of screening, if not delivered by a health care professional). This decision was made because we felt that it best resembles the types of interventions in the sample of Cochrane reviews.

### Items of interest and data extraction

We sought information on a variety of items relevant to four categories of interest:

1. the development of data extraction forms
2. the piloting of data extraction forms
3. the data extraction process
4. the use of software used in the review conduct

The list of items for each of these dimensions of interest was based on a previous project by us that reviewed methodological guidance on developing, piloting and performing data extraction in systematic reviews and modified for the purpose of this empirical analysis [23]. Further information on the rationale for each item is provided in the accompanying paper [23]. We did not look at items regarding risk of bias assessment, because this is often handled separately from data extraction.

We developed a standardized data extraction form to extract data related to these items using Microsoft Excel. This also included a comment field which was used to collect additional details and other items of interest. The first draft of the data extraction form was developed by RBB and reviewed by DP. The extraction form was then independently piloted by two reviewers (RBB and AW) using 6 systematic reviews and revised as needed. For this piloting process 3 Cochrane and 3 non-Cochrane reviews were used that were *not* part of the sample of reviews included in the analysis.

The piloting process was followed by a calibration exercise, in which two of us (RBB and AW) extracted data on a 20% random sample of reviews included in our analysis (15 Cochrane and 15 non-Cochrane reviews). This calibration exercise was used to further revise the codebook and improve consistency. Discrepancies in the data extracted for this 20% sample were documented and resolved by discussion or adjudication with a third author, if required (DP). After the calibration exercise for the Cochrane and non-Cochrane reviews were completed, one author extracted data from the remaining 80% of the included reviews (RBB). Major changes made to the data extraction form during the piloting and calibration process are reported in Additional file 3.

If information on specific items was reported in variable detail between protocols, study registry entries and the main publications of the reviews, we used the most comprehensive information. If the information provided was discordant, we used the information from the main publication. For example, if it was planned to involve three authors in data extraction according to a protocol, but due to a change in authors or author roles, only two performed the data extraction according to the final review, the latter information was used.

### Data analysis

We analysed data descriptively using frequencies, percentages, quartiles, and interquartile ranges (IQR). We used the total number of reviews as the denominator in our analysis unless stated otherwise.

We compared the methods reported in Cochrane versus non-Cochrane reviews using risk ratios and 95% confidence intervals for key items of interest and present these in forest plots. Data were analysed with JASP for Windows version 0.14.1 [24]. Risk ratios and forest plots were calculated and created in R version 4.1.0 using the meta package [25, 26].

## Results

We included 75 Cochrane reviews selected according to the methods described above. From the results of the Medline search, we consecutively screened a random sample of 190 titles and abstracts to reach a sample of at least 75 eligible non-Cochrane reviews. Raw agreement during title and abstract screening was 88% (167/190) with a Cohen’s kappa of 0.76 indicating “good” agreement.

We retrieved full texts of 104 potentially eligible non-Cochrane reviews and, where available, accompanying registrations and protocols. Raw agreement for assessment of eligibility based on these records was 88% (91/104) with a Cohen’s kappa of 0.69 indicating “good” agreement. Some cases of disagreements were due to poor reporting of the study selection process and different judgements. These cases were resolved by discussion with a third reviewer (DP).

From the 104 potentially eligible non-Cochrane reviews, 77 fulfilled our eligibility criteria and were included in the analysis. Thus, together with the sample of Cochrane reviews, we analysed a total of 152 systematic reviews. Together with the protocols and study registrations this resulted in a total of 256 reports. The study flow for the non-Cochrane reviews with reasons for exclusion is presented in Additional file 4. The list of included and excluded systematic reviews is available in the Open Science Framework (https://osf.io/ekt94/).

### Characteristics of the included systematic reviews

The reviews in our sample had a median of 5 authors (IQR: 3), with a slightly higher number of authors in the non-Cochrane reviews than the Cochrane reviews. None of the non-Cochrane reviews were updates of previous reviews, while this was the case for half of the Cochrane reviews. The median number of included studies was 11 (IQR: 15.25). Non-Cochrane reviews had included more studies than Cochrane reviews. Seven of the reviews in the sample had not included any studies, all of which were Cochrane reviews. A protocol was available for almost all Cochrane reviews, but only one of the non-Cochrane reviews. However, 47% (36/77) of the non-Cochrane reviews had been registered in PROSPERO.

The corresponding authors of the Cochrane reviews were most commonly affiliated with institutions in the UK (19/75 [25%]), while China was the predominant country in the sample of non-Cochrane reviews (21/77 [27%]). Authors affiliated with institutions in Canada and the USA were common among both groups. The Cochrane reviews also had several corresponding authors in Australia.

Overall, 76% of the reviews provided information on the number of reviewers involved in data extraction (116/152). Two authors were typically involved. Reviews in which two authors were involved had a median of 9 (IQR: 13) included studies, reviews in which more than two authors were involved a median of 14 (IQR: 36.75) included studies. Cochrane reviews with two data extractors had a median of 5 (IQR: 9.75) included studies, those with more than two extractors a median of 11 (IQR: 33). Non-Cochrane reviews with two data extractors had a median of 14 (IQR: 10) included studies, those with more than two extractors a median of 17 (IQR: 31). The data extraction form was published in 7% of the reviews (10/152).

The characteristics of the included systematic reviews are summarized in Table 1. Detailed information on the country of the first authors and the topic area based on International Classification of Disease (ICD) chapters are available in Additional file 5. This also includes the dates the Cochrane protocols were first published and the submission dates of the non-Cochrane reviews to PROSPERO.

**Table 1:** Characteristics of the included systematic reviews

	Total (*n* = 152)	Cochrane (*n* = 75)	Non-Cochrane (*n* = 77)
Number of authors	5 (4 to 7)	5 (4 to 6)	6 (4 to 9)
Update of a previous review	38 (25%)	38 (51%)	0 (0%)
Iteration of the update			
First	15 (10%)	15 (20%)	0 (0%)
Second	9 (6%)	9 (11%)	0 (0%)
Third	10 (7%)	10 (13%)	0 (0%)
Fourth	4 (3%)	4 (5%)	0 (0%)
Number of included studies	11 (4.75 to 20)	6 (3 to 18)	15 (9 to 21)
Review Registered	n/a	n/a	36 (47%)
Protocol available	68 (45%)	67 (89%)	1 (1%)
Reported adherence to PRISMA^a^	63 (41%)	1 (1%)	62 (81%)
Country of corresponding author			
Australia	8 (5%)	8 (11%)	0 (0%)
Canada	16 (11%)	9 (12%)	7 (9%)
China	22 (14%)	1 (1%)	21 (27%)
UK	26 (17%)	19 (25%)	7 (9%)
USA	18 (12%)	6 (8%)	12 (16%)
Other	62 (41%)	32 (43%)	30 (39%)
Funding source			
Non-commercial	98 (64%)	66 (88%)	32 (42%)
Commercial	1 (1%)	0 (0%)	1 (1%)
No funding	25 (16%)	3 (4%)	22 (29%)
Not reported	28 (18%)	6 (8%)	22 (29%)
Data extraction form published			
Yes	10 (7%)	7 (9%)	3 (4%)
No	142 (93%)	68 (91%)	74 (96%)
Number of reviewers involved in data extraction			
1	1 (1%)	0 (0%)	1 (1%)
2	74 (49%)	36 (48%)	38 (49%)
3	22 (14%)	18 (24%)	4 (5%)
4	8 (5%)	6 (8%)	2 (3%)
> 4	12 (8%)	9 (12%)	3 (4%)
Not reported	35 (23%)	6 (8%)	29 (38%)

Data given as number (percent) or median (25th to 75th percentile); percentages are rounded to the whole number; ^a^this excludes cases where only use of the PRISMA flow-chart is mentioned; *n/a* not applicable

### Preparation of data collection forms

In 32% of the reviews it was clear that the data extraction form was developed a priori (49/152). Furthermore, 51% of the reviews reported use of a standardised extraction form (77/152). In 24% of the Cochrane reviews, use of a newly developed form was reported (18/75), while 12% mentioned use of a generic form (9/75), and 7% of an adapted form (5/75). From the non-Cochrane reviews, 13% reported used of a newly developed form (10/77), while the remainder did not specify this. Methods to account for multiple study reports were reported in 31% of the reviews (47/152).

Only 2% of the reviews provided information on the number of authors that were involved in developing the data extraction form (3/152).

### Piloting of data collection forms

Piloting of the data extraction form was reported in 16% of the reviews (24/152). Five reviews stated how many reviewers were involved in piloting (5/152); this ranged from 1 to 6 authors.

Six Cochrane reviews and one non-Cochrane review reported the number of studies that were used to pilot the data extraction form (7/152): three reported piloting forms on two studies, one on three and one on five studies. The other two reviews reported use of “at least one” study without further specification. Three (Cochrane) reviews reported modifications from piloting the form (3/152): in two cases the authors reported “minor revisions”, while one review mentioned specific modifications to the collection of data on adverse events. None of the reviews reported whether data extractors had been trained.

### Performing data extraction

In total, 38% of the reviews (58/152) reported what data was extracted from the included studies, while 33% of them partially reported this information (59/152).

Data extraction procedures were reported in 88% of the reviews (133/152) with a variety of methods used. Data were extracted by two or more authors independently in 89% of the Cochrane (67/75) and 61% of the non-Cochrane reviews (47/77). A procedure where one author extracted data and another checked the data for accuracy was reported by 3% of the Cochrane (2/75) and 6% of the non-Cochrane reviews (5/77). Involvement of two reviewers without specification of the method was reported in 3% of the Cochrane reviews (2/75) and 5% of the non-Cochrane reviews (4/77). Few reviews used modifications of these procedures such as independent duplicate data extraction for outcome data and extraction by one author for non-outcome data (Additional file 6).

Procedures to resolve disagreements between data extractors were described by 70% of the reviews (107/152). The procedures were described as:

- discussion between the extractors with involvement of a third author if necessary (33/152)
- discussion between the extractors (32/152)
- arbitration (29/152)
- discussion or arbitration without further specification (13/152)

Three percent of the reviews that had included at least one study reported occurrences of disagreements between authors during data extraction (5/145). Four of these were Cochrane reviews.

Eight percent of the Cochrane reviews provided information on the expertise of the authors involved in data extraction (6/75): three reported content and methodological expertise, two content and one methodological expertise.

Incomplete or unpublished information from the included studies was sought in 68% of the reviews (103/152). The number of times study authors were contacted to acquire missing information or data was reported in 8% of the Cochrane (6/75) and 3% of the non-Cochrane reviews (2/77) and ranged from one to four times. One review stated that multiple attempts were made without further specifying this.

The use of procedures to avoid errors during data management was reported in 56% of the Cochrane (42/75) and 13% of the non-Cochrane reviews (10/77). Most commonly this included accuracy checks after entering data into software for analysis.

Finally, we noticed that 5/75 Cochrane reviews reported that the authors checked the included studies for retractions and errata (7%) and 1 non-Cochrane review reported checking for retractions (1%). This information was not included as an item in our data extraction form but collected informally in the comments column.

### Use of software to manage and analyse data

Software use to support study selection, data extraction, synthesis or grading of evidence was reported in 92% of the reviews (140/152). As we would expect, almost all Cochrane reviews reported use of Review Manger (RevMan) and GRADEpro GDT. Use of RevMan was also reported in 46% of the non-Cochrane reviews that mentioned software used for statistical analysis (27/59).

Few of the reviews in our sample reported use of software tools for study selection and data extraction. The most commonly reported tool for these purposes was Covidence, which was used for study selection in 12% of all reviews (18/152) and for data extraction in 6% of all reviews (9/152). Reporting of software use is summarized in Table 2.

**Table 2:** Use of software reported in the systematic reviews

	Total (*n* = 152)	Cochrane (*n* = 75)	Non-Cochrane (*n* = 77)
Software used for study selection			
Covidence	18 (12%)	16 (21%)	2 (3%)
DistillerSR	2 (1%)	0 (0%)	2 (3%)
EndNote	11 (7%)	5 (7%)	7 (9%)
Microsoft Excel	2 (1%)	1 (1%)	1 (1%)
Rayyan	2 (1%)	0 (0%)	2 (3%)
Other	3 (2%)	1 (1%)	2 (3%)
None reported	122 (80%)	58 (77%)	64 (83%)
Software used for data extraction			
Covidence	9 (6%)	8 (11%)	1 (1%)
DistillerSR	2 (1%)	0 (0%)	2 (3%)
Microsoft Excel	3 (2%)	2 (3%)	1 (1%)
Other	4 (3%)	1 (1%)	3 (4%)
None reported	133 (88%)	63 (84%)	70 (91%)
Software used for statistical analysis			
Comprehensive Meta-Analysis	6 (4%)	0 (0%)	6 (8%)
R	10 (7%)	0 (0%)	10 (13%)
RevMan	100 (66%)	73 (97%)	27 (35%)
Stata	18 (12%)	2 (3%)	16 (21%)
Trial Sequential Analysis (TSA)	4 (3%)	2 (3%)	2 (3%)
Other	7 (5%)	2 (3%)	5 (6%)
None reported	20 (13%)	2 (3%)	18 (23%)
Software used for grading of evidence			
GRADEpro GDT	72 (47%)	70 (93%)	2 (3%)
No software use reported	12 (8%)	0 (0%)	12 (16%)

Data given as number (percent); percentages rounded to the whole number; more than one software for the same purpose was used in some reviews

### Adherence to PRISMA reporting guidelines

Table 3 contrasts the reporting status in the reviews in our sample with related reporting guideline items from the PRISMA 2009 statement. The comparison shows that many reviews reported whether data were extracted independently and in duplicate, how disagreements between authors were resolved, who extracted data and whether additional information was sought from authors of the included studies. Other important aspects were, however, seldom reported. Only few reviews reported whether the data extraction form was piloted, what data were collected from the included studies and how the authors dealt with multiple study reports. None of the reviews provided information on reviewer training in data extraction and very few published the data extraction form that was used.

**Table 3:** Comparison of reporting with PRISMA 2009 reporting items

Suggestions in PRISMA 2009^**a**^	Total (***n*** = 152)	Cochrane (***n*** = 75)	Non-Cochrane (***n*** = 77)
Provide data extraction form	10 (7%)	7 (9%)	3 (4%)
Report use of a dedicated data extraction form	77 (51%)	49 (65%)	28 (36%)
Report whether the data extraction form was developed a priori	49 (32%)	33 (44%)	16 (21%)
Report whether the data extraction form was piloted	24 (16%)	14 (19%)	10 (13%)
Report whether a training exercise was undertaken	0 (0%)	0 (0%)	0 (0%)
Report the data collection process (in duplicate, independently or not)	133 (88%)	75 (100%)	58 (75%)
Report how disagreements were resolved	107 (70%)	70 (93%)	37 (48%)
Report who extracted the data	116 (76%)	69 (92%)	47 (61%)
Report the process used to obtain data from investigators^b^	103 (68%)	75 (100%)	28 (36%)
Report all variables for which data were collected^c^	58 (38%)	36 (48%)	22 (29%)
Report methods used to deal with multiple reports of the same study	47 (31%)	33 (44%)	14 (18%)

Data given as number (percent); percentages are rounded to the whole number; ^a^items refer to suggestions in the PRISMA explanation and elaboration document; ^b^we only documented whether this was done, irrespective of the methods; ^c^we considered this item to be fulfilled if information on at least one variable for each PICO component was provided

We note that some of the items in Table 3 come from recommendations in the PRISMA 2009 explanation and elaboration document, not just the checklist – and that some are considered minimum criteria, while others are suggestions for desired reporting [27]. We did not make a comparison with the 2020 PRISMA reporting guidelines, since these were just published [28].

### Comparison of Cochrane and non-Cochrane reviews

**Fig. 1:**
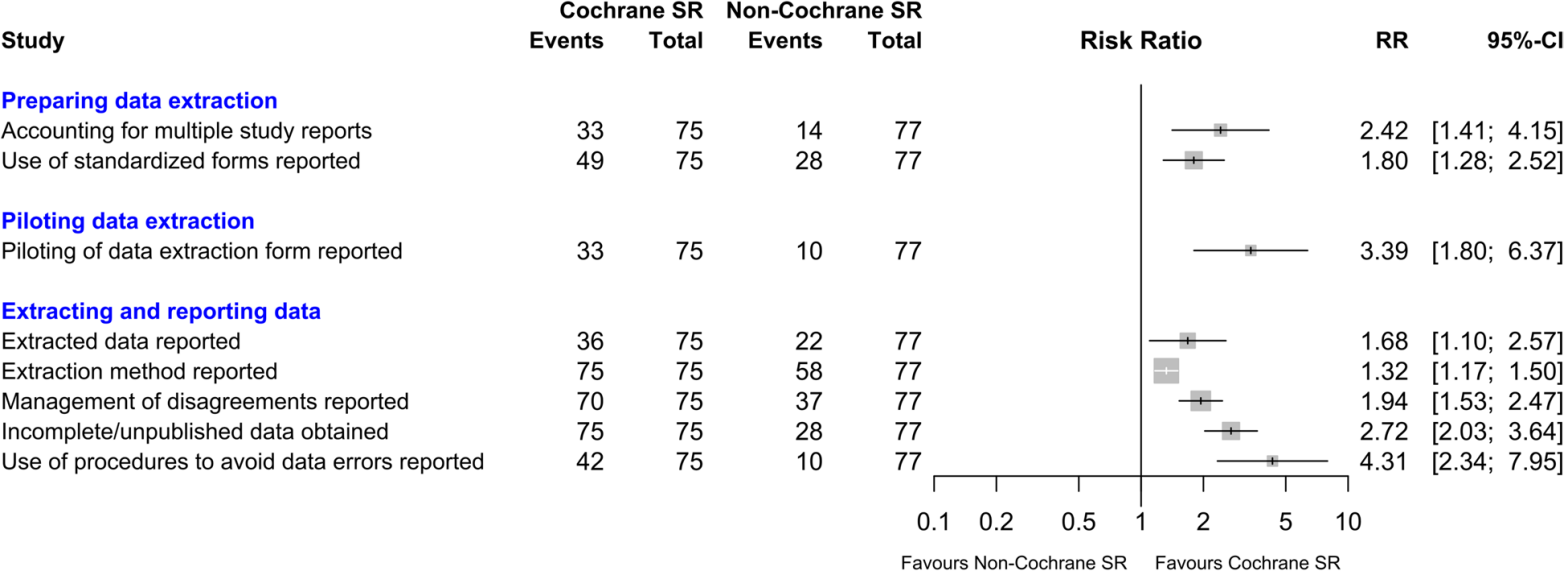
Comparison of Cochrane and non-Cochrane reviews

We calculated risk ratios to compare the reporting status of selected items for Cochrane and non-Cochrane reviews. These items were chosen because we considered them to be of particular interest to readers and of importance to the quality and usefulness of a systematic review [13]. The results are presented in Fig. 1 and show that Cochrane reviews were more likely to report all these items.

## Discussion

In summary our results show that reporting of the methods used to prepare and pilot data extraction forms in systematic reviews is limited. We would expect the reviews in our current sample to have a high reporting quality for items included in the 2009 PRISMA guidelines, considering that these have been available and promoted for 10 years now and are endorsed by many journals [29]. Furthermore, 81% of the authors of non-Cochrane reviews stated that they adhered to PRISMA. However, even aspects of data extraction strongly endorsed in PRISMA were not consistently reported. For example, a quarter of the non-Cochrane reviews did not report how data collection was performed (e.g., independently and in duplicate), less than half reported how disagreements were managed, only a third reported whether missing data was obtained and less than a fifth reported how multiple study reports were dealt with. Few systematic reviews provided precise information on what data were extracted from included studies.

It is unsurprising that the Cochrane reviews in our sample had a higher quality of reporting than the non-Cochrane reviews, which has been shown for other aspects of reporting previously [10]. Putting that aside, the non-Cochrane reviews in our sample may have actually had a higher need for sophisticated extraction and quality assurance methods due to larger authors groups and a higher number of included studies.

We acknowledge that some of the items we have looked at may be more of relevance to the authors conducting a review than the readers of the review – or that their relevance depends on factors such as the number of studies included in a review, the numbers of authors involved in collecting data, their experience and expertise, the topic and complexity of the review and the platform or tools used to conduct the review. For example, training of data collectors will be of particular relevance in mega-reviews with dozens or hundreds of included studies and large authors groups, particularly if not all data collectors are involved in developing and piloting the form [6, 23].

We also acknowledge that there is limited empirical evidence on the impact of several items that we have looked at. Thus, we cannot be sure whether, for example, piloting of data extraction forms or training of data extractors improves the quality of a review [4]. Evidence is available for some aspects of data extraction, however, including independent, duplicate extraction and adjudication [4, 30]. Furthermore, other items are important for transparency and to allow readers of reviews to make sense of the review and the underlying studies – and thus supported by a theoretical rationale [28].

### Use of software and automation tools

We noticed that the use of tools to support review conduct was rarely reported except for statistical software and RevMan, which is required in Cochrane reviews. This contrasts with many tools available: at time of writing, the Systematic Review Toolbox lists 220 software tools to support the development of systematic reviews in the healthcare field, 178 of which were listed as being free [31]. While the tools that were reported most often in our sample are fee-based, use of Covidence is free for use in Cochrane reviews and encouraged by Cochrane [32]. This likely explains why it was used 8 times as often in Cochrane reviews than non-Cochrane reviews.

The infrequent reporting of software tools is in contrast with a recent survey among systematic reviewers [33]. Here 89% of the respondents had used such tools previously and more than 50% had used them in at least half of their systematic reviews. The most common applications were at the screening stage (79%), at the data extraction stage including risk of bias assessment (51%) and for data synthesis (46%). This difference may be due to the self-selected nature of the survey respondents. That said, we cannot rule out that the use of tools was not fully reported in our sample. The selection of tools used by the reviewers in the abovementioned survey are in line with our results, however: Covidence, RevMan and GRADE Pro GDT were the most widespread. The study also explored reasons for low uptake of such tools with lack of knowledge, costs and too much effort to learn being reported most often.

### Implications for research

A possible explanation for the limited reporting of many aspects of preparing, piloting and performing data extraction is limited and scattered guidance. A recent methodological review of systematic review handbooks, HTA method documents, textbooks and peer reviewed papers showed that only few of these documents provided comprehensive recommendations [23]. Thus, we believe it would be helpful to develop clearer guidance for authors of systematic reviews. This could include a minimum set of criteria that should be considered to ensure a high quality of data collection, taking into account the needs of different reviews based on review size, team size, review complexity, platform and software use as well as available resources. For example, few reviews in our sample reported modifications of the standard data collection process such as independent and duplicate data extraction for results and outcome data and verification for non-outcome data such as baseline data. This is surprising considering that such modification of the data extraction process can save resources without compromising quality [3]. In developing such recommendations, relevant previous literature should be taken into account [3–5, 13, 23, 28, 30, 33].

Another possible explanation is that the PRISMA 2009 guidelines were somewhat vague regarding the information that should be reported on some aspects of data extraction. This has been addressed in the PRISMA 2020 update which now distinguishes between essential elements and additional elements and presents recommendations in a clear bullet point format [28].

### Suggestions for systematic reviewers

It is beyond the scope of our analysis to provide comprehensive guidance on data collection methods. That said, we have made some general suggestions including pointers to further literature. These are presented in Table 4.

**Table 4:** Suggestions for planning and performing data extraction in systematic reviews

Issues to consider	References
Consider the specific review requirements early in the process including review complexity, size, resources, and experience and expertise of data collectors.	[6, 34]
Develop a thoughtful data collection form with clear instructions.	[6, 13]
Consider using existing and proven forms and adapt them as required, if available.	[23, 35]
Consider specific data collection requirements for more complex methods such as individual patient data or network meta-analyses and make use of available guidance for such situations.	[36]
Pilot data collection forms using a purposive sample of studies in light of the review specifics. This could, for example, include a mix of well and less well reported studies and different study designs or outcomes.	[13, 23]
Consider the merits and downsides of different extraction methods in the light of resources requirements, risk of errors and the severity of possible errors.	[3, 4, 30]
Be cognisant and reflective of the intricacies of coding such as stability, accuracy, reproducibility, and effects of framing, learning and fatigue.	[34]
When resolving disagreements between reviewers, make sure that a fair procedure is in place to avoid decision making simply based on seniority, experience, or power.	[30, 37]
Take advantage of software that can help to support workflows, keep a paper trail, and reduce risk of extraction errors.	[3, 6, 31, 38]

In addition to the considerations on the data extraction process, we suggest that systematic reviewers publish their data extraction forms as a supplement or in a repository. This would greatly increase transparency, make the reviews more applicable and allow assessment by readers by providing direct insights into the data that were collected and the coding methods used.

### Limitations

Our analysis has some limitations. First, about half of the Cochrane reviews were updates of previous reviews. While review methods evolve, authors may stick with methods used in previous iterations of their reviews for pragmatic reasons or consistency, for example. Thus, our results may underestimate the quality of reporting of methods in newly initiated Cochrane reviews. While we also used the publication date as an entry criterion for non-Cochrane reviews, most of these were submitted to PROSERO in 2019 or 2020 and can be considered current.

Secondly, we used a rough classification for the item on whether the reviews reported which data were extracted from the included studies. Furthermore, Cochrane reviews typically report the PICO criteria with much more detail than non-Cochrane reviews and hereby provide a better indication of the collected data. Therefore, the comparison for this item needs to be interpreted with some caution.

Thirdly, we restricted our analysis to systematic reviews of medical interventions in humans. Thus, they may not be representative of other review types. Finally, while we were careful in developing, piloting and calibrating our data collection process, data extraction was performed by a single author for 80% of the reviews in our sample, which may have introduced some errors or oversights.

## Conclusion

We show that reporting of the methods used for data extraction in systematic reviews is currently limited. We believe that publishing of data extraction forms alongside systematic reviews would greatly increase transparency, make systematic reviews more applicable and allow assessment by readers. More and clearer guidance for systematic reviewers taking into account the requirements of different reviews seems desirable. Our results on software use can serve as a baseline to assess the uptake of tools to assist data extraction and other steps in the conduct of systematic reviews in future analyses.

## Supplementary Information

**Additional file 1.** Search Strategy.

**Additional file 2.** Study selection sheet.

**Additional file 3.** Changes to extraction form after piloting and calibration.

**Additional file 4.** PRISMA flow-chart.

**Additional file 5.** Additional sample characteristics.

**Additional file 6.** Modifications of data extraction methods used in reviews.

## Data Availability

The datasets generated and analysed are available in the Open Science Framework (https://osf.io/ekt94/).

## Abbreviations

CDSR: Cochrane Databases of Systematic Reviews
GRADEpro GDT: Grading of Recommendations Assessment, Development and Evaluation Guideline Development Tool
HTA: Health Technology Assessment
PICO: Population Intervention Comparison Outcome
PRISMA: Preferred Reporting Items for Systematic Reviews and Meta-Analyses
PROSPERO: International Prospective Register of Systematic Reviews
RevMan: Review Manager
