# Supplementary material for: Reporting of methods to prepare, pilot and perform data extraction in systematic reviews: analysis of a sample of 152 Cochrane and non-Cochrane reviews
Source: BMC Med Res Methodol. 2021 Nov 6;21:240. doi: 10.1186/s12874-021-01438-z (PMC8571672; doi:10.1186/s12874-021-01438-z)
Supplement: Supplementary file 1 — Additional file 1. Search Strategy. [file 12874_2021_1438_MOESM1_ESM.docx]

**Additional file 1: search strategy for non-Cochrane systematic review sample**

**Search strategies for literature search**

**Medline search string (via PubMed)**

#1 MEDLINE[Title/Abstract] OR (systematic[Title/Abstract] AND review[Title/Abstract]) OR meta-analysis[Publication Type]

#2 (clinical[Title/Abstract] AND trial[Title/Abstract]) OR clinical trials as topic[MeSH Terms] OR clinical trial[Publication Type] OR random*[Title/Abstract] OR random allocation[MeSH Terms] OR therapeutic use[MeSH Subheading]

#3 #1 AND #2

#4 animals[mh] NOT humans[mh]

#5 #3 NOT #4

#6 covid-19 OR 2019-nCoV OR "2019 novel coronavirus" OR SARS-CoV-2 OR "severe acute respiratory syndrome coronavirus 2"[Supplementary Concept]

#7 #5 NOT #6

#8 #7 AND english[la]

#9 #8 AND 2020/05/01:2020/06/30[dp]
