# Supplementary material for: Reporting of methods to prepare, pilot and perform data extraction in systematic reviews: analysis of a sample of 152 Cochrane and non-Cochrane reviews
Source: BMC Med Res Methodol. 2021 Nov 6;21:240. doi: 10.1186/s12874-021-01438-z (PMC8571672; doi:10.1186/s12874-021-01438-z)
Supplement: Supplementary file 3 — Additional file 3. Changes to extraction form after piloting and calibration. [file 12874_2021_1438_MOESM3_ESM.docx]

**Additional file 3: Piloting and calibration procedure and major changes to data extraction form after piloting and calibration**

**Procedure for piloting the data extraction sheet:**

- Piloting conducted with reviews not included in this study.
- Sample: 3 Cochrane and 3 non-Cochrane reviews
- Conducted by two reviewers independently and in duplicate.
- Modifications and disagreements discussed with a third reviewer.

**The following changes were made after piloting the sheet:**

- We added an extra item to the extraction form that indicates whether the review authors have reported which information they will extract or have extracted from the included studies. The rationale for this was that readers of a review can be more confident that information missing in the details of the characteristics of included studies is not due to lack of reporting of the review authors, but rather the underlying study reports. Provision of information on the PICO was not considered for the reporting of this item, although inevitably, there is some overlap (depending on details of reporting of the PICO in the review). Three response options were considered for coding: yes, partially, no.
- We added a response option for the item on the method used to extract the data from study reports to incorporate cases where an independent parallel extraction was conducted for outcome data, while non-outcome data such as study characteristics was extracted by one person only.
- We changed the wording and coding for the item on use of automation tools to software in general and differentiated the response option to “yes, for data extraction” and “yes, but not for data extraction”.

**Procedures for calibration of the data extraction:**

- Calibration conducted with reviews included in this study.
- Sample: 15 Cochrane and 15 non-Cochrane reviews
- Conducted by two reviewers independently and in duplicate.
- Modifications and disagreements discussed with a third reviewer.
- Data extractions that had already occurred were re-checked after the changes that were made during calibration.

**The following changes were made after calibration:**

- We added an “unclear” response option to the item "linking of multiple study reports", because we noticed that in some reviews it was unclear whether the authors refer to duplicates or multiple publications of the same study.
- We added an “unclear” response option to the item “unpublished data obtained”, because we noticed that in some cases the authors did not report this but excluded studies because of missing data – which can be the result of either not attempting to retrieve additional data or of unsuccessful attempts to retrieve additional data.
- We specified the coding options for several items, particularly:
  - What was considered to count as a standardized extraction form.
  - How the methods used for resolving disagreements between reviewers was coded.
  - How we defined and coded a pre-developed data extraction form.
- We decided to collect information on the use of software reported in the systematic reviews irrespective of the purpose of use (e.g. supporting the review workflow, study selection, data extraction, statistical analysis, grading evidence or writing the review). This decision was made to capture fully the use of software tools in reviews considering the recent interest and relevance of automation tools for systematic review conduct.
