# Supplementary material for: Reporting of methods to prepare, pilot and perform data extraction in systematic reviews: analysis of a sample of 152 Cochrane and non-Cochrane reviews
Source: BMC Med Res Methodol. 2021 Nov 6;21:240. doi: 10.1186/s12874-021-01438-z (PMC8571672; doi:10.1186/s12874-021-01438-z)
Supplement: Supplementary file 4 — Additional file 4. PRISMA flow-chart. [file 12874_2021_1438_MOESM4_ESM.docx]

**Additional file 4: PRISMA flow diagram**

**Identification of Systematic Reviews from Medline search**

Total (N = 2742)

- Medline records excluded due to ineligibility based on title and abstract (n = 86*)
- Records excluded after random sample was full (n = 2656)

Records identified from Medline (N= 2846)

Reports sought for retrieval

- Journal publications of potentially eligible systematic reviews (N = 104)

Linked records retrieved (N=42)

- PROSPERO records (n = 41**)
- Published protocols (n = 1)

Systematic reviews assessed for eligibility based on reports (N = 146)

- Journal publications (n = 104)
- PROSPERO records (n = 41)
- Published protocols (n = 1)

Reports included (N = 114)

- Systematic reviews included (n = 77)
- PROSPERO records included (n = 36)
- Protocols included (n = 1)

Systematic reviews excluded (N = 27)

- Research question not defined (n = 2)
- Eligibility criteria not reported (n = 5)
- Search sources not reported (n = 1)
- Selection methods not reported (n = 12)
- Overview of reviews (n = 1)
- No critical appraisal (n = 6)

PROSPERO records excluded (N = 5)

* The reasons for exclusions during title and abstract screening were:

- Animal review: 2
- Diagnostic review: 6
- Duplicates of Cochrane reviews: 3
- Methodological review: 7
- Mixed review type: 10
- Not a medical intervention: 10
- Not a systematic review: 10
- Prevalence review: 4
- Prognosis/risk factor review: 18
- Protocol: 14
- Scoping review: 2

**One review reported registration in PROSPERO but did not provide a record number. We were unable to identify the record based on the author names and review topic and author contact was unsuccessful.

From: Page MJ, McKenzie JE, Bossuyt PM, Boutron I, Hoffmann TC, Mulrow CD, et al. The PRISMA 2020 statement: an updated guideline for reporting systematic reviews. BMJ 2021;372:n71. doi: 10.1136/bmj.n71

For more information, visit: <http://www.prisma-statement.org/>
