# Supplementary material for: Reporting of methods to prepare, pilot and perform data extraction in systematic reviews: analysis of a sample of 152 Cochrane and non-Cochrane reviews
Source: BMC Med Res Methodol. 2021 Nov 6;21:240. doi: 10.1186/s12874-021-01438-z (PMC8571672; doi:10.1186/s12874-021-01438-z)
Supplement: Supplementary file 5 — Additional file 5. Additional sample characteristics. [file 12874_2021_1438_MOESM5_ESM.docx]

**Additional file 5: Additional sample characteristics**

|  |  | Total (n=152) | | Cochrane (n = 75) | | Non-Cochrane (n = 77) | |
| --- | --- | --- | --- | --- | --- | --- | --- |
|  |  | n | % | n | % | n | % |
| Year of publication | 2020 | 146 | 96% | 75 | 100% | 71 | 92% |
|  | 2019 | 5 | 3% | 0 | 0% | 5 | 6% |
|  | 2018 | 1 | 1% | 0 | 0% | 1 | <1% |
| Year of publication of protocol/ submission to PROSPERO | 2020 | 2 | 1% | 0 | 0% | 2 | 3% |
|  | 2019 | 20 | 13% | 3 | 4% | 17 | 22% |
|  | 2018 | 22 | 14% | 9 | 12% | 13 | 17% |
|  | 2017 | 12 | 8% | 11 | 15% | 1 | 1% |
|  | 2016 | 8 | 5% | 5 | 7% | 3 | 4% |
|  | 2015 | 5 | 3% | 5 | 7% | 0 | 0% |
|  | 2014 | 4 | 3% | 4 | 5% | 0 | 0% |
|  | 2013 | 1 | 1% | 1 | 1% | 0 | 0% |
|  | 2012 | 6 | 4% | 6 | 8% | 0 | 0% |
|  | 2011 | 2 | 1% | 2 | 3% | 0 | 0% |
|  | 2010 | 6 | 4% | 6 | 8% | 0 | 0% |
|  | before 2010 | 15 | 10% | 15 | 16% | 0 | 0% |
|  | not available | 49 | 32% | 8 | 11% | 41 | 53% |
| Country of corresponding author | Argentina | 1 | 1% | 1 | <1% | 0 | 0% |
|  | Australia | 8 | 5% | 8 | 11% | 0 | 0% |
|  | Bahrain | 1 | 1% | 1 | <1% | 0 | 0% |
|  | Brazil | 5 | 3% | 1 | <1% | 4 | 5% |
|  | Canada | 16 | 11% | 9 | 12% | 7 | 9% |
|  | China | 22 | 14% | 1 | <1% | 21 | 27% |
|  | Denmark | 2 | 1% | 2 | 3% | 0 | 0% |
|  | Ecuador | 2 | 1% | 2 | 3% | 0 | 0% |
|  | France | 1 | 1% | 1 | <1% | 0 | 0% |
|  | Germany | 4 | 3% | 1 | <1% | 3 | 4% |
|  | Greece | 1 | 1% | 0 | 0% | 1 | <1% |
|  | Hungary | 1 | 1% | 1 | <1% | 0 | 0% |
|  | India | 1 | 1% | 0 | 0% | 1 | <1% |
|  | Indonesia | 1 | 1% | 1 | <1% | 0 | 0% |
|  | Iran | 3 | 2% | 1 | <1% | 2 | 3% |
|  | Ireland | 2 | 1% | 2 | 3% | 0 | 0% |
|  | Italy | 6 | 4% | 3 | 4% | 3 | 4% |
|  | Lithuania | 1 | 1% | 0 | 0% | 1 | <1% |
|  | Malaysia | 3 | 2% | 3 | 4% | 0 | 0% |
|  | Mexico | 1 | 1% | 1 | <1% | 0 | 0% |
|  | Netherlands | 5 | 3% | 3 | 4% | 2 | 3% |
|  | New Zealand | 4 | 3% | 3 | 4% | 1 | <1% |
|  | Norway | 1 | 1% | 0 | 0% | 1 | <1% |
|  | Phillippines | 1 | 1% | 1 | <1% | 0 | 0% |
|  | Saudia Arabia | 1 | 1% | 0 | 0% | 1 | <1% |
|  | South Africa | 4 | 3% | 3 | 4% | 1 | <1% |
|  | South Korea | 3 | 2% | 0 | 0% | 3 | 4% |
|  | Spain | 4 | 3% | 0 | 0% | 4 | 5% |
|  | Syria | 1 | 1% | 0 | 0% | 1 | 1% |
|  | Taiwan | 1 | 1% | 0 | 0% | 1 | 1% |
|  | Thailand | 1 | 1% | 1 | <1% | 0 | 0% |
|  | UK | 26 | 17% | 19 | 25% | 7 | 9% |
|  | USA | 18 | 12% | 6 | 8% | 12 | 16% |
|  | Summe | 152 |  | 75 |  | 77 |  |
| Topic area (ICD chapter) | A | 0 | 0% | 0 | 0% | 0 | 0% |
|  | C | 15 | 10% | 6 | 8% | 9 | 12% |
|  | D | 5 | 3% | 4 | 5% | 1 | 1% |
|  | E | 19 | 13% | 12 | 16% | 7 | 9% |
|  | F | 15 | 10% | 4 | 5% | 11 | 14% |
|  | G | 8 | 5% | 4 | 5% | 4 | 5% |
|  | H | 7 | 5% | 7 | 9% | 0 | 0% |
|  | I | 22 | 14% | 12 | 16% | 10 | 13% |
|  | J | 3 | 2% | 3 | 4% | 0 | 0% |
|  | K | 16 | 11% | 3 | 4% | 13 | 17% |
|  | L | 6 | 4% | 3 | 4% | 3 | 4% |
|  | M | 7 | 5% | 0 | 0% | 7 | 9% |
|  | N | 7 | 5% | 3 | 4% | 4 | 5% |
|  | O | 5 | 3% | 5 | 7% | 0 | 0% |
|  | P | 4 | 3% | 3 | 4% | 1 | 1% |
|  | Q | 2 | 1% | 1 | 1% | 1 | 1% |
|  | R | 4 | 3% | 2 | 3% | 2 | 3% |
|  | S/T | 3 | 2% | 1 | 1% | 2 | 3% |
|  | V/Y | 0 | 0% | 0 | 0% | 0 | 0% |
|  | Z | 4 | 3% | 2 | 3% | 2 | 3% |
|  | U | 0 | 0% | 0 | 0% | 0 | 0% |
