# Supplementary material for: Reporting of methods to prepare, pilot and perform data extraction in systematic reviews: analysis of a sample of 152 Cochrane and non-Cochrane reviews
Source: BMC Med Res Methodol. 2021 Nov 6;21:240. doi: 10.1186/s12874-021-01438-z (PMC8571672; doi:10.1186/s12874-021-01438-z)
Supplement: Supplementary file 6 — Additional file 6. Modifications of data extraction methods used in reviews. [file 12874_2021_1438_MOESM6_ESM.docx]

**Supplementary file 6: Modifications of the data extraction methods used in systematic reviews**

Most of the reviews in our sample reported use of independent and duplicate data extraction. Modifications of the data extraction procedure that we noted, were:

- independent duplicate data extraction for outcome data and extraction by one author for non-outcome data (used in one Cochrane review)
- extraction of data by one author and verification for accuracy on a sample of included studies by a second author (used in one Cochrane review)
- independent duplicate data extraction for outcome data and extraction of study characteristics by one author with verification by a second author for a sample of studies (used in two Cochrane reviews)
- extraction of data by one author and verification for accuracy for outcome data on all studies and verification for accuracy of study characteristics on a sample of studies (used in one non-Cochrane review)
